# COVID-19 pandemic and the social determinants of health

**DOI:** 10.3389/fepid.2023.1139371

**Published:** 2023-05-31

**Authors:** John E. Meador, Wesley James, Joseph Branson, Jonathan Bennett, Karen Matthews

**Affiliations:** ^1^ Delta Health Alliance, Stoneville, MS, United States; ^2^ Center for Community Research and Evaluation, University of Memphis, Memphis, TN, United States

**Keywords:** COVID-19 vaccination, interventions, minority, community, health, rural

## Abstract

Hesitancy to receive a COVID-19 vaccination across sub-groups within the US population contributed to higher illness rates and deaths. Specifically, minority groups and those living in rural and remote areas are more vaccine-hesitant populations known to suffer from higher disparities in health. Identifying successful and replicable approaches to promoting vaccination within these subpopulations is critical to ensuring vaccination rates can be maximized in these vulnerable groups. In this paper, we present findings from the Mississippi Recognizing Important Vaccine & Education Resources (RIVERs) project, a multi-state effort to spread accurate information related to COVID-19 vaccinations using a variety of community and media-based methods as well as provide vaccinations. Vaccination rates for Black people in Mississippi exceeded those of White people, likely due to the concerted effort of regional health and community organizations. Propensity score matching is performed to test intervention styles using spatial and temporal data related to approximately 7,000 events across Mississippi and parts of Tennessee and publicly available data on vaccination rates and socio-economic data. We demonstrate that vaccination rates within the vulnerable groups may be closely related to misinformation being spread through local social networks and that interventions carried out by local leaders with high levels of local social capital are best at quashing misinformation at the local level. We recommend that policymakers consider the importance of local efforts as an effective tool in increasing vaccination rates in future pandemics.

## 1. Introduction

**Figure 1:**
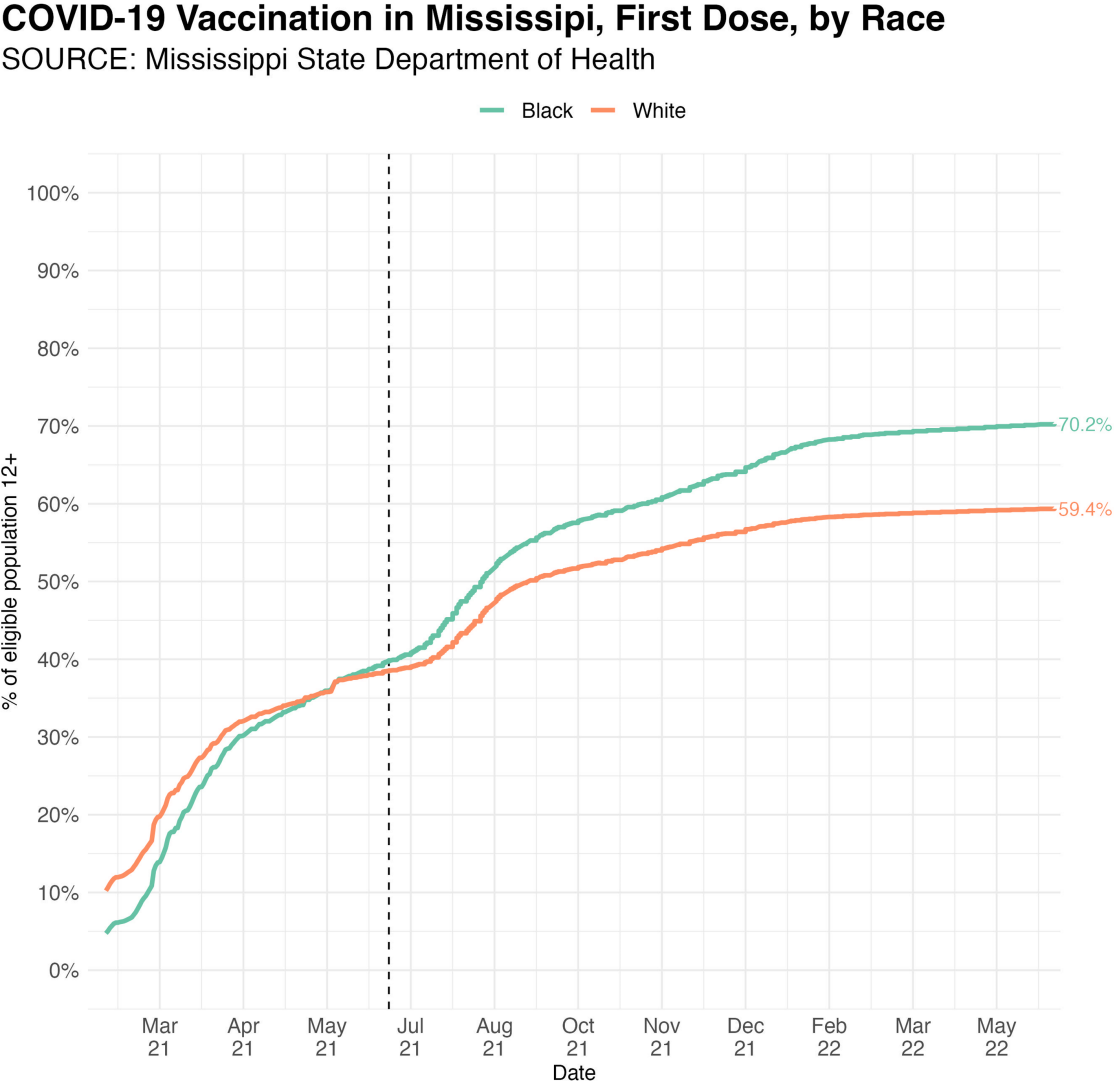
Daily cumulative growth in COVID-19 vaccinations by race in Mississippi. Black people vaccination rates overtake white people vaccination rates in summer 2021. Black dotted line highlights date of first RIVERs intervention in Mississippi.

The COVID-19 pandemic and the hesitancy of some population groups to get vaccinated have highlighted the importance of large public campaigns to promote vaccine uptake in the U.S. Public vaccination campaigns can take a variety of approaches, and different approaches can be tailored to fit regional or culturally unique subpopulations. Predicting which campaigns work best across regions and subpopulations will help ensure vaccination uptake occurs effectively. This paper presents findings from an analysis of a state-wide effort in Mississippi known as the RIVERs program. Despite Mississippi residents’ high poverty rates and history of institutional distrust in government and healthcare, they increased their national ranking in vaccinations. The increase is partly due to an increase in African American vaccination rates, which overtook white Mississippians’ vaccination rates over time (see Figure 1).

### 1.1. Vaccinations and race

The impact of COVID-19 is not spread evenly across racial groups in the U.S. For example, studies conducted early in the pandemic noted that Non-Hispanic Black people had been negatively impacted at a notably higher rate when compared to other racial and ethnic groups. For instance, Price-Haywood et al. (1) racial differences in hospitalizations of COVID-19 patients in Louisiana and found that Black people comprised about 80% of the hospitalizations when only about 31% of them routinely received care from the hospital group. Gold et al. (2) found similar results in their study of COVID patients in March of 2020—over 80% of COVID-19 patients were non-Hispanic Black people, a number substantially higher than the proportion of non-Hispanic Black people living in the study area. A further study conducted in Chicago in the winter of 2020 found similar results; multivariate analysis of patient data on hospital admissions due to COVID-19 linked with social and demographic data revealed that being a Black person and older were the only statistically significant indicator of hospital admission (3). Racial differences in deaths due to COVID-19 were also identified during early periods of the pandemic, with Black Americans dying from the disease at disproportionally higher rates in the US (4).

A critical question from studies conducted early in the COVID-19 pandemic was to what extent exogenous social and economic characteristics impact the likelihood that a person ends up in the hospital due to contracting COVID-19. Raifman and Raifman (5) used data from the 2018 Risk Factor Surveillance System and modeled population risk factors for becoming ill with COVID-19 at a national level. They found that 33% of Black Americans were at a higher risk of becoming very ill with COVID-19 compared to only 27% of White Americans. Only American Indians were at higher risk than Black Americans, at 42%. The authors also found that low-income households were at higher risk and that the compounding nature of poverty, access to care, and lifestyle characteristics may impact sub-sets of the population at greater levels.

The reality that people living under undue economic burden suffer disproportionally from ill health is not new. Black Americans have an overall shorter life expectancy than non-Hispanic White Americans. Some researchers theorize that the compounding effect of poor mental health coping strategies, environmental, social, and economic inequalities explain the drastic difference in life expectancies (6). Khazanchi et al. (7) argue that the disproportional number of minorities entering the hospital due to COVID-19 and the lack of any statistical differences in proportional outcomes between races once admitted to the hospital support their theory that “racism, not race” is to blame for the current inequities.

### 1.2. Geographic inequalities

People living in rural areas of the US face unique social and economic challenges that can make coping with the pandemic harder than their urban-dwelling counterparts. The rural economy is driven primarily by agriculture, forestry, and tourism. Jobs in these sectors are primarily seasonal, and many are informal (8), meaning that many job opportunities include any form of associated health insurance plan. Rural areas have consistently higher poverty rates than metro areas (9). Furthermore, people living in rural areas are often geographically isolated and have issues accessing health care services, lack reliable high-speed broadband access, and report high stigma associated with care, especially mental health services (10). The onset of the COVID-19 pandemic exacerbates the social and economic hardships in rural areas in the US. For instance, Mueller et al. (11) found that the economic shutdown and subsequent re-opening policies fueled an unemployment rate of about 9.74% in rural areas, compared to 7.40% nationally. These compiling factors are not dispersed equally across racial groups in rural areas, and there is evidence that racial differences in rural areas are disproportionally impacted. For instance, Henning-Smith et al. (12) found that rural counties in the US with the highest levels of non-Hispanic Black people or American Indian/Alaska Native (AI/AN) see the most significant proportion of premature death rates in the country.

There is clear evidence that the COVID-19 pandemic and subsequent lockdowns disproportionately impact minority populations and those living in rural areas of the US. Indeed, evidence also supports that minority populations living in rural areas experience a compounding effect and are at an even greater risk of ill effects of the virus and the social and economic disruptions caused by its reaction. The vaccine rollout, which began in late 2020, could reduce risks associated with contracting COVID-19 and reduce the racial inequalities in current COVID-19 death rates. However, recent research from residents in Arkansas suggests that vaccine hesitancy may be highest in non-Hispanic Black people due to fear and general mistrust (13).

### 1.3. RIVERs program & vaccination interventions

The RIVERs program is a consortium effort led by Delta Health Alliance in Stoneville, MS, to promote the uptake of COVID-19 vaccination in Mississippi and Tennessee. Program delivery occurred through various community and media interventions from June 2020 through May 2022. Program staff involved in community interventions participated in training on the science around vaccination and its impact on COVID-19 and training on working with local residents who may be vaccine-hesitant.

There are 25 categories of intervention types (see Table 1 for a complete list), each with a varying degree of community involvement. For example, some interventions took place at local libraries and involved discussion groups between program staff and local community members on the merits of vaccination; other interventions included mass media efforts featuring state-born celebrities that aired weekly on TV and social media platforms.

**Table 1:** Recoded intervention categories and methods.

**Recoded category**	**Original category**
Vaccine access	This is a vaccine delivery site; Transportation/getting to a vaccine delivery site
Traditional media	A radio spot; A tv spot
Visiting community	Visiting a community-based recreation center; Visiting a church, temple, or other religious site/building; Visiting a local school, college, or a community learning center; Visiting an lgbtq community resource center; A community fair or event
Digital impression	Jumbotron; A social media site
Personal communication	Text message; Door-to-door outreach; Door hangers; A training session; Other form of in-person interaction not listed here; ^a telephone call; Focus group; Email
Virtual	A virtual town hall; A community website, blog, or web-based tool about covid-19 vaccines; A webinar
Information flyers	Flyers; Billboards or other types of posters/signs around the community; Educational and/or informational fliers about covid-19 vaccines; ^general information on covid-19 vaccines; Mail

## 2. Method

A two-step research design is used to determine which categories of community interventions are associated with more people becoming vaccinated. The first step is to create a control group of counties that received no program interventions. The second step is to determine which interventions are associated with significant changes in vaccination rates between each treatment county and its matched control county.

### 2.1. Creating a control group

Matching is often used in the social sciences to identify sub-populations that can act as a comparable control group in observational studies. Using matching algorithms to create control groups is preferred over identifying a purely randomized control group in observational studies because it allows for any biased or spurious covariates to be controlled for during the matching process (14). This approach is particularly useful to this research due to the previously detailed social and environmental factors that are believed to influence people to receive a COVID-19 vaccination. The *MatchIt* package (15) in the R programming language (16) is used to perform matching. The Mahalanobis distance approach is used, rather than propensity scores, due to relatively few covariates and all covariate’s approximate normal distribution (17).

#### 2.1.1. Identifying co-variates

Treatment counties include only counties in Mississippi that received some community intervention during the RIVERs program. For data consistency, the five counties receiving Tennessee interventions are not included.

All counties in the US except those in Mississippi and the five program counties in Tennessee are included in the pool of potential matches. Variable selection is based on previously mentioned literature and all data included are from the US Census Bureau published in 2020 except for the percent of the county that is classified as rural. The US Census has not yet published data on percent of each county classified as rural as of this publication. Therefore, percent of county classified as rural according to the US Census in 2010. Covariate variables are:

∙ Percent of county classified as African American alone;
∙ Median household income by county;
∙ Percent of county aged 65 or older;
∙ County population; and,
∙ Percent of county classified as rural.

### 2.2. Identifying intervention impact

The RIVERs program conducted about 7,200 interventions from July 2, 2021, to May 21, 2022. Interventions were categorized according to a list of 25 potential delivery methods. An intervention could be categorized into more than one category. Each intervention category has been recoded to fit the following categories based on the type of activity: visiting community, personal communication, info-flyers, digital impression, vaccine access, traditional media, and virtual.

Vaccination totals by county come from the Centers for Disease Control and Prevention’s website and data portal. Daily vaccination totals were merged with daily intervention data for each county. The date of the first and last intervention for each county; the total number of each intervention type were then calculated for each county; then, the change in vaccination totals for each treatment county and its matching control county is calculated. Finally, the difference between a change in the vaccination total of each treatment county and its matching control county is calculated. This data is then merged with the data of covariates mentioned above.

The resulting database contains a column indicating the difference in treatment and control vaccination totals and count data on the total number of interventions for each category. An Ordinary Least Squares (OLS) model is produced using difference in vaccination totals between treatment and control counties as the dependent variable. Equation 1 details the full model, and results are presented in Table 3.

(1) $$\begin{array}{rl} \Delta \; & \text{Vaccination}\\ = & \alpha +\beta_{1}(\text{Household income})+\beta_{2}(\text{Black people alone})\\ & +\beta_{3}(\text{Percent county rural})+\beta_{4}(\text{Digital impression})\\ & +\beta_{5}(\text{Flyers})+\beta_{6}(\text{Personal communication})\\ & +\beta_{7}(\text{Virtual meeting})+\beta_{8}(\text{Visit community center})\\ & +\beta_{9}(\text{Traditional media})+\epsilon \end{array}$$

## 3. Results

### 3.1. Bivariate correlations

Bivariate Pearson correlations are found in Table 2. All covariates are statistically associated vaccination numbers in the treatment counties. Percent of Black residents, and the percent of a county with a bachelors degree or higher are positively correlated with vaccination numbers while household income, age and percent rural are negatively associated with vaccination numbers. Percent rural is also negatively associated with vaccination numbers in control counties as well.

**Table 2:** Correlations: changes in vaccination and socio-demograpnics for treatment and control groups.

**Variable**	**Treatment**	**Control**
Percent of county that classifed as black people alone	0.274${\;}^{*}$	0.107
Median household income by county	−0.269${\;}^{*}$	0.008
Percent of county aged 65 and older	−0.230${\;}^{*}$	−0.183
Percent of county with a bachelors degree or higher	0.277${\;}^{*}$	0.068
Percent of county classified as rural	−0.372${\;}^{***}$	−0.277${\;}^{*}$

${\;}^{*}p\geq 0.1$; ${\;}^{**}p\geq 0.05$; ${\;}^{***}p\geq 0.01$.

### 3.2. Matching results

Matched results have a standardized mean difference (SMD) close to zero, indicating a good balance in covariates. All but one matched county comes from a southern state, and about 42.7% of matched counties come states that border Mississippi.

### 3.3. OLS results

Examing multicollinearity revealed a high degree of correlation (−0.560, Pearson) between Percent African American and Household income for treatment counties. This was only slightly less for control counties (−0.538, Pearson). We therefore removed this variable from the multi-variate model. OLS model results, shown in Table 3 has an adjusted R-squared value of 0.4064, suggesting a good fit relative to other social science research models (18).

**Table 3:** OLS: DP is the difference in change in vaccination of treatment and control counties.

**Term**	**Estimate**	**Std_Error**	**Statistic**	**Prob**
Intecept	−13,470.13	3371.464	−3.995	0.0${\;}^{***}$
Percent African American	7,330.09	3682.115	1.991	0.0503${\;}^{*}$
Percent county rural	10,705.07	3123.975	3.427	0.001${\;}^{***}$
Digital impressions	−1.37	0.367	−3.728	0.000${\;}^{***}$
Flyers	0.00	0.120	−0.016	0.987
In-person communication	0.30	0.151	1.989	0.0505${\;}^{*}$
Virtual meeting	−0.23	1.004	−0.225	0.823
Visit community center	−0.48	0.281	−1.690	0.095${\;}^{*}$
Traditional media	0.10	0.049	2.135	0.036${\;}^{**}$

$R^{2}$ 0.465; Adj. $R^{2}$ 0.4064.

${\;}^{*}p\geq 0.1$; ${\;}^{**}p\geq 0.05$; ${\;}^{***}p\geq 0.01$.


$$\begin{array}{rl} \Delta \;\text{Vaccination} & =-13470.13+7330.09(\text{Percent African American})\\ & \;+10705.07(\text{Percent county rural})\\ & \;-1.37(\text{Digital Impressions})+0(\text{Flyers})\\ & \;+0.3(\text{In-person Communication})\\ & \;-0.23(\text{Virtual Meeting})\\ & \;-0.48(\text{Visit Community Center})\\ & \;+0.1(\text{Traditional Media}) \end{array}$$

Model coefficients suggest that personal communication and traditional media approaches have a positive and statistically significant association with increased numbers of people vaccinated in treatment counties compared to control counties. Furthermore, higher digital impressions are statistically associated with lower numbers of vaccinations in treatment counties compared to control counties. No other variables were found to be statistically significant.

## 4. Discussion

For the past two years, COVID-19 represents the third leading cause of death in the United States, with more than 350,000 deaths per year (CDC FastStats). COVID-19 related illnesses, hospitalizations, and deaths are not distributed evenly. Pre-existing social and economic disparities in these outcomes have been exacerbated, as evidenced in a plethora of studies in the academic literature and popular media (cite several of the studies you have already cited maybe even a couple new ones). In nearly every measurable health, social, and economic outcome, Mississippi ranks at or near the bottom, making the state the ultimate petri dish for negative COVID-19 related outcomes. Regarding specific COVID-19 outcomes, in 2020, Mississippi had the highest per capita death rate in the nation at 1,138.7 deaths per 100,000 (KFF). In comparison, the U.S. average was 835.4 per 100,000. At the beginning of the RIVERs project, Mississippi had the lowest vaccination rate in the county, with less than one-third of residents having received at least one dose of the vaccine. The U.S., however, exhibited a rate of 46.2% (KFF). Although the state has experienced its share of struggles, the RIVERs project has demonstrated a remarkable success for the most vulnerable citizens of a state rife with every layer of disadvantage.

Current data shows that Mississippi is the only state in the U.S. with high levels of rural poverty where the Black population has higher rates of COVID-19 vaccinations than the white population. At the close of the RIVERs project in April of 2022, 69.6% of Black people had received their first vaccination, compared to only 58.9% of White people. In addition, Black people continued to widen the gap of becoming fully vaccinated over White peple, a 9.0% difference (62.4% vs 53.4%). The improvement in vaccine uptake appears to coincide with the start of the RIVERs program. Readers should note that we cannot definitively assign causality due to the myriad policy changes and community efforts unrelated to RIVERs that likely contributed to the increased uptake of vaccinations.

However, these results are evidence of the power of community engagement and optimization of local leadership being leveraged to educate community members and successfully counteract misinformation, while of course also protecting the most vulnerable citizens. The concerted efforts in Mississippi are proven to be effective given the lack of success in other states and counties with comparable social and economic circumstances. Candidly, the positive effects of the RIVERs project in Mississippi should serve as a model for positive public health intervention outcomes in other communities across the nation both now and in the future. Success in a place with every conceivable built-in disadvantage in society suggests that success should be achievable elsewhere as well. Policy makers, health practitioners, and local leaders should consider greater emphasis and investment in community level initiatives in future public health crises, either as a primary mechanism or in conjunction with other proven interventions and methodologies.

## 5. Limitations

Due to data being aggregated to the county-level, a sample of 82 yields a corresponding low statistical power. This is a limitation of our study and should be considered in replicated studies or drawing policy conclusions. Readers should note that alpha levels for significant testing begin at 0.10.

This study also lacks important contextual considerations as controlling covariates, mainly the likely impact that vaccination access (out-with of that offered by the RIVERs program as an event) and any local policies regarding vaccination status. Future studies may benefit from including local access to vaccinations and policies to better parse the impact of local vaccination efforts.

## Data Availability

The raw data supporting the conclusions of this article will be made available by the authors, without undue reservation.
